# Prevalence of long‐term mechanical insufflation‐exsufflation in children with neurological conditions: a population‐based study

**DOI:** 10.1111/dmcn.14797

**Published:** 2021-01-03

**Authors:** Brit Hov, Tiina Andersen, Michel Toussaint, Maria Vollsæter, Ingvild B Mikalsen, Solfrid Indrekvam, Vegard Hovland

**Affiliations:** ^1^ Division of Paediatric and Adolescent Medicine Oslo University Hospital Oslo Norway; ^2^ Institute of Clinical Medicine University of Oslo Oslo Norway; ^3^ Norwegian Advisory Unit on Long‐term Mechanical Ventilation Thoracic Department Haukeland University Hospital Bergen Norway; ^4^ Physiotherapy Department Haukeland University Hospital Bergen Norway; ^5^ Centre for Neuromuscular Disorders and Home Mechanical Ventilation UZ Brussel‐Inkendaal Vlezenbeek Belgium; ^6^ Department of Paediatrics Haukeland University Hospitals Bergen Norway; ^7^ Department of Clinical Science University of Bergen Bergen Norway; ^8^ Department of Paediatric Medicine Stavanger University Hospital HF Stavanger Norway

## Abstract

**Aim:**

To determine the prevalence of long‐term mechanical insufflation‐exsufflation (MI‐E) and concomitant mechanical ventilation in children with neurological conditions, with reported reasons behind the initiation of treatment.

**Method:**

This was a population‐based, cross‐sectional study using Norwegian national registries and a questionnaire.

**Results:**

In total, 114 of 19 264 children with a neurological condition had an MI‐E device. Seventy‐three of 103 eligible children (31 females, 42 males), median (min–max) age of 10 years 1 month (1y 5mo–17y 10mo), reported their MI‐E treatment initiation. Overall, 76% reported airway clearance as the main reason to start long‐term MI‐E. A prophylactic use was mainly reported by children with neuromuscular disorders (NMDs). Prevalence and age at initiation differed by diagnosis. In spinal muscular atrophy and muscular dystrophies, MI‐E use was reported in 34% and 7% of children, of whom 83% and 57% respectively received ventilator support. One‐third of the MI‐E users were children with central nervous system (CNS) conditions, such as cerebral palsy and degenerative disorders, and ventilator support was provided in 31%. The overall use of concomitant ventilatory support among the long‐term MI‐E users was 56%.

**Interpretation:**

The prevalence of MI‐E in a neuropaediatric population was 6 per 1000, with two‐thirds having NMDs and one‐third having conditions of the CNS. The decision to initiate MI‐E in children with neurological conditions relies on clinical judgment.

**What this paper adds:**

- The prevalence and age at initiation of mechanical insufflation/exsufflation (MI‐E) differed between diagnoses.
- MI‐E was most commonly used in spinal muscular atrophy, where it generally coincided with ventilatory support.
- One‐third of MI‐E devices were given to children with central nervous system conditions, and one‐third also received ventilatory support.

## What this paper adds

- The prevalence and age at initiation of mechanical insufflation/exsufflation (MI‐E) differed between diagnoses.
- MI‐E was most commonly used in spinal muscular atrophy, where it generally coincided with ventilatory support.
- One‐third of MI‐E devices were given to children with central nervous system conditions, and one‐third also received ventilatory support.

This article is commented on by Langdon and Cooper on pages 494–495 of this issue.

**Abbreviations**
LTMV: Long‐term mechanical ventilation
MI‐E: Mechanical insufflation‐exsufflation
NMD: Neuromuscular disorder
SMA: Spinal muscular atrophy

In children with neurological conditions, respiratory complications can be life threatening. Compromised respiration and cough may cause accumulation of airway secretion, possibly leading to pneumonia and respiratory failure.^1^, ^2^, ^3^ The impaired cough may be assisted mechanically using mechanical insufflation‐exsufflation (MI‐E) that augments the inspiratory volume and expiratory flow necessary in an efficient cough.^4^

MI‐E is well established in neuromuscular disorders (NMDs), especially for those with severe muscular weakness,^5^, ^6^, ^7^ preventing airway infections and hospitalizations.^3^, ^8^ Daily use supports airway clearance and promotes deep inspirations, especially in young children who do not benefit from other airway clearance techniques.^5^, ^6^, ^7^, ^9^, ^10^, ^11^ Recently, MI‐E treatment has been suggested for children with impaired cough due to quadriplegic cerebral palsy (CP),^12^, ^13^ as severe motor impairment may lead to compromised airway clearance and hospitalizations.^1^, ^2^, ^14^, ^15^, ^16^, ^17^

To help decide if MI‐E is indicated, Peak Cough Flow is the preferred measurement of cough capacity.^18^ However, being effort dependent, it has major limitations in children. Thus, initiating paediatric MI‐E treatment has largely been dependent upon clinical judgment. Clinical indications for MI‐E in children with muscle weakness are recurrent, prolonged respiratory infections and weak cough and voice.^5^, ^6^, ^7^ Since 2001, the Norwegian health authorities have provided publicly funded MI‐E devices when prescribed by a specialized physician, independent of area of residence or socioeconomic status, and without financial restriction.

Information on children with a neurological diagnosis who have an MI‐E device is scarce. Our aim was to determine the overall prevalence of long‐term MI‐E in the Norwegian paediatric population with a neurological diagnosis and to identify the clinical assessments and reported reasons behind initiation of the MI‐E treatment. The secondary aim was to describe the concomitant use of long‐term mechanical ventilation (LTMV) support in children equipped with an MI‐E device.

## Method

The present study was a population‐based, cross‐sectional study approved by the regional committee for medical research ethics and the institutions data board (https://clinicaltrials.gov/ identifier NCT03831568). Written informed consent was obtained for participation and publication of data. The reporting was guided by the STROBE statement.^19^

### Study population

The population of interest, termed ‘the neuropaediatric population’, was defined as children (<18y) with a main or bi‐diagnosis in the International Classification of Disease (ICD‐10)^20^ Chapter G ‘Diseases of the nervous system’ or a main diagnosis in Chapter E 70–90 ‘Metabolic disorders’.

The children were divided into two groups based on their main diagnosis: (1) NMD including (ICD‐10 code) spinal muscular atrophy (SMA) (G12) and muscular dystrophies/myotonies (G70–72); (2) conditions originating from the central nervous system (CNS) including encephalitis (G00–09), degenerative disorders in the CNS (G30–32), CP (G80–83), and other CNS disorders (G90–99). To maintain anonymity in rare conditions (<5), children with a main diagnosis classified as a metabolic disorder (E70–90, *n*=4) or peripheral nerve disease (G60–64, *n*=1) were classified as having a CNS disorder as they had a CNS condition as a bi‐diagnosis. Detailed descriptions of diagnostic coding and ICD‐10 codes are provided in Appendix S1 and Table S1 (online supporting information).

### Data sources and the outcomes

Data were collected from national registries and a questionnaire answered by children/parents equipped with an MI‐E device for long‐term use. Registry 1: Departments for Medical Home‐Care Equipment located at 18 hospitals provided records of all Norwegian children holding a MI‐E device. Registry 2: The Norwegian Patient Registry, a national registry including specialized health‐service activity data, provided: (1) anonymous data of age and regional residency of all children with any main or bi‐diagnosis defined as the neuropaediatric population, independent of MI‐E use. (2) Anonymous data of diagnosis, age, and regional residency for all children supplied with a MI‐E device for long‐term use. (3) The specific main and bi‐diagnosis of children returning consent form. Registry 3: Statistics Norway provided the overall count of the Norwegian population by regions, by 1st July 2017. Registry 4: The Norwegian registry for LTMV included descriptions of ventilator treatment (data extracted: 15th Oct 2018), including: interface (mask or tracheostomy), ventilation mode (continuous positive airway pressure/bi‐level/respirator), clinics (hypoventilation, hypoxaemia, symptoms), duration of daily usage, and supplementary equipment. Questionnaire: The questionnaire, developed in cooperation with MI‐E user‐representatives, surveyed children/parents about the initiation of MI‐E treatment (age, reasons behind initiating treatment), assessments performed (chest X‐ray, lung‐function, cough ability measurements, maximal mouth pressures), and the location and clinician that initiated the treatment.

### The study progress

The study commenced in two phases as illustrated in Figure S1 (online supporting information). Phase 1 assessed the prevalence of children with an MI‐E device in the Norwegian neuropaediatric population. All 18 hospital departments supplying medical equipment for long‐term use (Registry 1) listed children born after 1st February 1999 who had an MI‐E device. Children deceased or without a diagnosis in the neuropaediatric defining ICD‐10 codes (Fig. S1) were excluded by The Norwegian Patient Registry (Registry 2). Thus, the identified study population 1 was used for calculation of point prevalence of MI‐E users.

In Phase 2, paediatric pulmonologists and physiotherapists in six regional hospitals responsible for the children’s pulmonary care, confirmed the accuracy of study population 1, ensured that it was ethically appropriate to send a letter of invitation for participation, and excluded children using the exclusion criteria shown in Figure S1. Eligible children were included in study population 2 and were invited to answer the questionnaire. Concomitant ventilatory support was assessed using data from the Norwegian registry for LTMV (Registry 4).

### Statistical analysis

**Figure 1:**
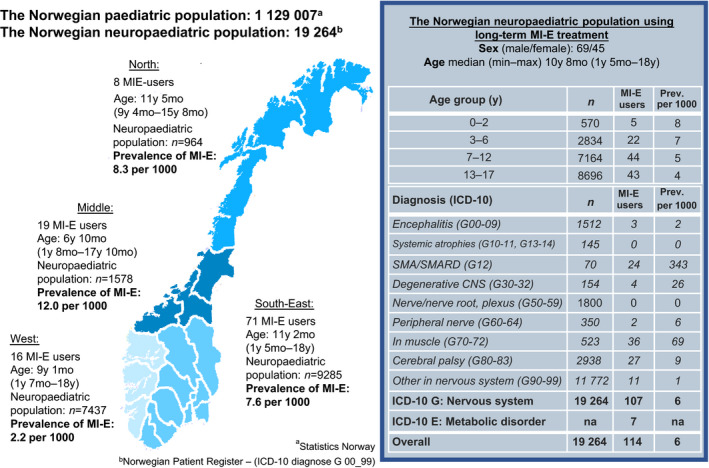
Prevalence of long‐term mechanical insufflation‐exsufflation (MI‐E) in a neuropaediatric population by diagnosis, age, and regional residency. ICD, International Classification of Disease; SMA, spinal muscular atrophy; SMARD, spinal muscular atrophy with respiratory distress.

The point prevalence of MI‐E users was calculated as the number of living children aged <18 years equipped with an MI‐E device on the prevalence day (15th August 2017) as the numerator and the neuropaediatric population as the denominator. The prevalence was estimated both for the overall neuropaediatric population and for subsets of children by ICD‐10 codes, age, and regional residency (Fig. 1). Age at prevalence day and age at first establishment of the primary data‐source (1st April 2017) were used when reporting age for study populations 1 and 2 respectively.

All continuous data proved to deviate significantly from normal, thus, distribution of all continuous variables is summarized as medians (min–max), and categorical data as counts and percentages. The denominator, differing according to data‐sources and missing variables (e.g., from incomplete questionnaires), is provided in Tables 1 and 2. Non‐parametric tests were used to compare differences between two groups via Wilcoxon rank‐sum tests and between three groups using the Kruskal–Wallis equality‐of‐populations rank test. Pearson’s *χ* ^2^ tests for contingency tables were applied to analyse differences between proportions. If cells had an expected value of <5, Fischer’s exact test was used. *p*‐values of <0.05 were considered significant. Statistical analyses were performed using STATA SE software, version 15.0 (StataCorp LLC, College Station, TX, USA).

**Table 1:** Demographics and clinical characteristics of Norwegian children using MI‐E (study population 2) according to diagnosis group

Variables	Study population 2, *n*	Overall (*n*=73)	Neuromuscular disorders (*n*=47)		Central nervous system conditions (*n*=26)	*p*
			Spinal muscular atrophy (*n=*24)	Muscular dystrophies/ myopathies (*n*=23)		
Age, y:mo	73	10:1 (1:5–17:10)	9:11 (1:5–17:10)	11:4 (4:3–17:1)	9:3 (1:7–16:9)	0.544
Age at MI‐E initiation,^a^ y	70	5 (0–15)	3 (0–14)	5 (1–14)	6 (0–15)	**0.041**
MI‐E use duration at study,^a^ y:mo	70	2:6 (0–14)	3:1 (0–14)	3 (0–10)	2 (0–12)	0.12
Sex						
Male	73	42 (58)	13 (54)	17 (74)	12 (46)	
Female		31 (42)	11 (46)	6 (26)	14 (54)	
Comorbidity						
Sleep disorder	73	28 (38)	9 (38)	8 (35)	11 (42)	0.859
Epilepsy		19 (26)	1 (4)	3 (13)	15 (58)	**<0.001** ^c^
Additional treatment						
CPAP for airway clearance^a^	69	15 (22)	0	2 (9)	13 (50)	**<0.001** ^c^
Long‐term mechanical ventilation^b^	41	41 (56)	20 (83)	13 (57)	8 (31)	**<0.001**

Data are median (min–max) or *n* (%). Age is calculated at identification of the primary data‐source. Comorbidities: sleep disorders includes International Classification of Diseas (ICD)‐10 G47.0–G47.9; epilepsy includes ICD‐10 G40.0–G40.9. *p*‐value refers to the difference between diagnosis groups calculated by Kruskal–Wallis rank test when continuous data or χ^2^ (Pearson) test when categorical data. *p*<0.05 marked in bold. Data source is Norwegian Patient Registry unless ^a^data from questionnaire or ^b^data from Norwegian registry for long‐term mechanical ventilation. ^c^Fisher’s exact test. MI‐E, mechanical insufflation‐exsufflation; CPAP, continuous positive airway pressure.

**Table 2:** The sub‐group in study population 2 using concomitant long‐term mechanical ventilation and mechanical insufflation‐exsufflation, according to diagnosis group

Variable	Overall			CNS	*p*
		SMA	Muscular dystrophies/myopathies		
Study population 2	73	24	23	26	
LTMV users	41 (56)	20 (83)	13 (57)	8 (31)	<0.001
Sex					
Male	24 (59)	11 (55)	8 (62)	5 (63)	**0.025** ^a^
Female	17 (41)	9 (45)	5 (38)	3 (38)	
Ventilatory mode					
CPAP	5 (12)	0	1 (8)	4 (50)	**0.002** ^a^
Bi‐level PAP	33 (81)	19 (95)	11 (84)	3 (38)	
Respirator	3 (7)	1 (5)	1 (8)	1 (12)	
Interface					
Tracheostomy	2 (5)	0	1 (8)	1 (13)	0.256^a^
Mask	39 (95)	20 (100)	12 (92)	7 (88)	
Daily usage					
<12h	32 (78)	16 (80)	11 (85)	5 (63)	0.558^a^
>12h	9 (22)	4 (20)	2 (15)	3 (38)	
Supportive equipment					
Oxygen supplement	9 (22)	5 (25)	1 (8)	3 (38)	0.292^a^
Humidifier	37 (90)	19 (95)	10 (77)	8 (100)	0.306^a^
Back‐up ventilator	19 (46)	8 (38)	6 (46)	5 (63)	0.344^a^
Suction device	20 (49)	8 (38)	6 (46)	6 (75)	0.234^a^
Oximeter	15 (37)	9 (45)	2 (15)	8 (100)	0.095^a^
Hypoventilation (before), *n*	28	13	10	5	0.755^a^
Yes	12	4	6	2	
No	16	9	4	3	
Hypoxemia (SpO_2_ <90%), *n*	18	9	5	4	0.966
Yes	13	6	4	3	
No	5	3	1	1	
Symptoms (before) yes/no, *n*					
Respiratory tract infections	33; 27/6	15; 13/2	11; 10/1	7; 4/3	0.251^a^
Insomnia	27; 7/26	12; 3/9	9; 2/7	6; 2/4	1.000^a^
Dyspnoea	25; 5/20	11; 1/4	9; 3/6	5; 1/4	0.469^a^
Fatigue	27; 7/20	12; 3/9	10; 3/7	5; 1/4	1.000^a^
At LTMV initiation					
Age, median (min–max)	2 (0–18)	2:6 (0–13)	2 (0–18)	1 (0–12)	0.544
Hospitalization days, median (min–max)	7 (1–199)	3 (1–83)	22 (2–105)	56 (5–199)	**0.016**
Nocturnal capillary PCO_2_ kPh, *n*; median (min–max)	30; 5.5 (4.4–8.9)	17; 5.3 (4.4–8.4)	7; 6.6 (4.4–7.1)	6; 5.6 (4.4–8.9)	0.257
Nocturnal average SpO_2_, % *n*; median (min–max)	34; 96 (91–99)	17; 96 (92.5–99)	10; 97 (94.6–99)	7; 96 (91–97)	0.103
Part of night with SpO_2_ <90%, *n*; median (min–max)	18; 1 (0–36)	9; 2.6 (0–5)	5; 0.3 (0–36)	4; 0.8 (0–10)	0.098

Data are *n* (%) unless otherwise stated. The clinical characteristics of the subgroup using long‐term ventilation (LTMV) support according to diagnosis group. *p*‐value refers to the difference between diagnosis groups calculated by Kruskal–Wallis equality‐of‐populations rank test when continuous data or χ^2^ test for contingency tables when categorical data. *p*<0.05 marked in bold.

^a^ Fisher’s exact test. SMA, spinal muscular atrophy; CNS, central nervous system; CPAP, continuous positive airway pressure; PAP, positive airway pressure.

## Results

In total, 114 of the 19 264 children (45 females, 69 males; median [min–max] age 10y 8mo [1y 5mo–18y]) in the neuropaediatric population were equipped with an MI‐E device for long‐term use, giving an overall point prevalence of 6 per 1000 (study population 1, Fig. S1). The prevalence of MI‐E users in the 114 children differed by diagnosis, age, and region of residency (Fig. 1). The highest prevalence was found in SMA, where 24 of 70 children (34%) had an MI‐E device, ranging from 24% to 40% among the health regions (

Fig. S2, online supporting information).

Of the 114 children in study population 1, 11 were excluded according to the criteria shown in Figure S1, leaving 103 eligible children for inclusion in the second study‐phase (study population 2, Fig. S1), where 73 (71%) children consented to participate. The demographics and clinical characteristics of responders are provided in Table 1, and diagnosis, age, and regional residency of non‐responders in Figure S1.

Child/parent reported data regarding initiation of the MI‐E treatment are shown in Table S2 (online supporting information). Clincal signs of impaired mucus clearence, such as weak cough, inability to clear airway mucus, and/or frequent respiratory infections, were reported as reasons behind initiation in 53 of 70 (76%) patients. Prevention of airway infections, without present signs of mucus retention, was reported in 17 (24%) children. Such prophylactic MI‐E use was more commonly reported among children with NMDs (*n*=15) compared to those with CNS conditions (*n*=2, *p*=0.011) (Table S2).

At MI‐E commencement, 33 children/parents reported various assessments being performed. By diagnosis (NMD/CNS) these were chest X‐ray (11/6), lung function measurement (19/4), Peak Cough Flow (11/1), and maximal mouth pressure (5/1) (Fig. S3, online supporting information). Of 36 children not recalling any assessment before initiation, 22 were <6 years and nine had a CNS condition.

Forty‐two per cent reported that MI‐E commenced ‘during acute admissions due to respiratory infections’, 31% ‘at admissions for other reasons’, and 20% ‘electively in the outpatient clinic’. MI‐E treatment was titrated and patients were instructed in use of the equipment by physiotherapists (64%), physicians (6%), or teams (30%) (Table S2). Regional differences in the child/parent reported reason behind initiation were not found (Table S3, online supporting information).

LTMV was used together with MI‐E in 41 (56%) of the 73 children. Ventilator support was more common in children with NMDs, used by 83% and 57% of children with SMA and muscular dystrophies/myotonies respectively, compared to 31% of children with CNS conditions (*p*<0.001). Peak Cough Flow measurements were only reported in children >8 years of age, with a median (min–max) value of 147l/min (41–274l/min) in 9 of 41 children using LTMV. Median duration of hospitalizations at LTMV initiation in children with CNS conditions, muscular dystrophy/myotonies, and SMA were 56 (5–199), 22 (2–105), and three (1–83) days respectively (*p*=0.016). Ventilator support was delivered non‐invasively in 39 of 41 (95%) children. Bi‐level modes were used in 30 of 33 (91%) of children with NMDs and in 3 of 8 (38%) children with CNS conditions. In CNS conditions, continuous positive airway pressure was used in 4 of 8 (50%) children. The presence of hypercapnia/hypoxia and symptoms by diagnosis group are reported in Table 2.

Intermittent continuous positive airway pressure (airway clearance purposes) was reported by 15 of 69 (22%) children, more commonly in CNS conditions (50%) compared to NMDs (4%) (*p*<0.001) (Table 1).

## Discussion

In this Norwegian, nationwide, population‐based study, 6 per 1000 children in the neuropaediatric population were equipped with an MI‐E device for long‐term use. Age at initiation and prevalence differed by diagnosis. Two‐thirds of the MI‐E devices were given to children with NMD and one‐third to children with CNS conditions. The highest prevalence was found in SMA, where one in three children used an MI/E. Symptomatic affected airway clearance was the most frequently reported incentive to start long‐term MI/E, whereas prophylactic use to prevent respiratory infections was mainly reported in children with NMDs. More than half of the children using MI‐E used ventilator support.

We studied the prevalence and not the incidence of long‐term MI‐E, both being affected by the mortality rate of the diseases. The mortality was high in the present study as 12 of 124 children died during a period of less than a year before invitation (Fig. S1), in line with the mortality rates reported by others.^11^ The mortality rate and the time lag between identifying the population and the data sampling, possibly resulted in missing children who initiated MI‐E in this period. The prevalence calculated may, therefore, be falsely low, particularly in children with a diagnosis associated with high mortality. Additionally, a single‐centre study reported an increase in MI‐E use over the last years,^8^ mainly explained by more aggressive supportive treatment in children with SMA. The same may be true in Norway, suggesting the prevalence of long‐term MI‐E in 2020 is probably higher than in the present study which used 2017 data. Furthermore, the slightly higher median age in the non‐responders may result in an underestimation of age at initiation and total user‐years. One could question whether the primary data source is truly comprehensive, as it does not assess private purchase of the equipment. However, as the costly equipment is provided for free by the Norwegian government if prescribed by a physician, private purchases seem unlikely.

The child/parents reported the reasons behind commencing MI‐E treatment retrospectively, with a risk of recall bias, and these should not be mistaken as the medically founded indications. Although the questionnaire was developed in cooperation with MI‐E users, it was not formally validated. Nevertheless, we aimed to describe rather than test associations, thus the requirements for internal validity are less rigid. Our descriptive findings may generate further hypotheses and aid clinical judgments regarding long‐term MI‐E treatment and concomitant ventilator support.

To our knowledge, this is the first study describing the prevalence of long‐term MI‐E users by diagnosis and age in a nationwide paediatric population. Comparison of results with other national populations are thus difficult. However, long‐term MI‐E use was most frequent in SMA and muscular dystrophies/myotonies, diagnoses often reported in other MI‐E studies.^10^, ^11^ The present study did not only target NMDs, but a comprehensive neuropaediatric population. It may, thus, add insight to an expanded group of diagnoses where long‐term MI‐E is used in children, namely conditions originating in the CNS. Both in degenerative disorders in the CNS and CP, MI‐E was prevalent in 3% and 1% of children respectively. In fact, one‐third of all devices belonged to children with CNS conditions, suggesting that MI‐E had been judged beneficial for airway clearance in these children. This is supported by Siriwat et al. who found MI‐E beneficial in reducing therapy time in hospitalized children with CP presenting with respiratory infections and atelectasis.^12^ This finding is relevant, since respiratory complications are the most common cause of emergency admissions in children with CP, and severe CP is associated with lower pulmonary capacity, severe respiratory muscle weakness, and more hospitalizations compared to mild CP.^15^, ^17^

In the present study, children with CNS conditions reported total ‘user‐years’ of MI‐E of only 2 years, lower than ‘user‐years’ reported by children with NMDs. This may suggest that long‐term MI‐E is an emerging, non‐evidence‐based practice in children with CNS conditions, where respiratory complications are a significant cause of morbidity.^1^, ^2^

Notably, even in a country with equal access to health services, we found regional differences in the prevalence of long‐term MI‐E. This may not only be due to the rare heterogeneous diagnoses, but also to local preferences and underlines the importance of clinical guidelines to aid decision making on who may benefit from MI‐E treatment. Nevertheless, the high proportion of MI‐E users among children classified with typical NMDs indicates that the Norwegian practice follows international recommendations for airway clearance in NMD.^5^, ^6^, ^7^

Only 12 children reported measurement of Peak Cough Flow, the most common assessment of cough capacity.^18^ However, a high proportion of participants were <6 years, limiting the possibility of performing voluntary measurements. Additionally, developmental disorders may hamper cooperation with such measurements. This underlines the difficulty in obtaining an objective measure to aid the decision whether MI‐E is indicated. Thus, the decision remains a clinical judgement in many cases.

Preventive MI‐E treatment was reported primarily in children with NMDs. This may be the result of well‐described disease pathways and treatments protocols available for NMDs, recommending MI‐E at an early age.^7^, ^9^ MI‐E may be implemented to prevent and treat respiratory events, but also to familiarize the child with the device, as well as maintaining chest wall compliance.^11^ In CP, initiation of MI‐E cannot rely only on the diagnosis, and preventive use of MI‐E in this group of children appears untargeted. However, other techniques (e.g., air‐stacking using glossopharyngeal breathing) are limited in young or non‐cooperative children. Thus, MI‐E might be indicated more frequently in young children compared to older and cooperating children, who have a larger selection of treatment choices.

The reported reasons behind starting MI‐E treatment were the presence of a clinical problem affecting airway clearance, including a weak and ineffective cough or a problem with recurrent respiratory infections. In the diverse group of CNS conditions, recurrent respiratory infections are frequently reported.^1^, ^2^, ^14^, ^16^, ^17^ In the present study, half of the children with CNS conditions used intermittent continuous positive airway pressure for airway clearance purposes, supporting the position that airway secretion may be an unsolved problem. Respiratory function is related to the level of motor impairment in CP,^15^ and MI‐E may be considered in children with the most severe impairment, who present a weak and ineffective cough, cumulating secretions in large airways that results in recurrent respiratory tract infection. However, it is important to rule out and treat possible causes of excessive airway mucus, such as aspiration due to dysphagia or gastro‐oesophageal reflux.^1^, ^21^ In CNS conditions, unlike typical NMDs, a weak cough may not always be the obvious clinical sign indicating the need for cough augmentation. The cough strength may be sufficient to transport mucus and the cough deficiency may instead be related to abnormal control of swallowing and an impaired cough reflex,^21^ resulting in missing cough attempts despite audible accumulated airway secretions that further result in retention and respiratory infections. Given the heterogenic nature of many of the disorders reported in the present paper, we strongly emphasize that a clinical trial to evaluate the response should be mandatory before equipping any child with an MI‐E device, independent of diagnosis. Additionally, the child/parent perception of the MI‐E use should be explored and included in the decision process, aiming for truly family‐centred care.

MI‐E treatment is considered an important adjunct to LTMV^7^, ^9^ and 56% of children using MI‐E also received ventilator support. The concurrent use was more common in SMA, found in 83% of children, compared to in CNS conditions, where LTMV was reported in only 31%. Reporting from a specialized LTMV healthcare centre, Chatwin and Simonds^11^ described as many as 96% of the individuals received both long‐term MI‐E and LTMV. However, in that study more than 70% of participants were older than 18 years, and included diagnoses not prevalent in a paediatric population, thus making comparison difficult.

The Norwegian registry for LTMV reported a coverage of 82% in the paediatric population (SI, personal communication, annual report 2017). The missing 18% may introduce a falsely low number of MI‐E users having ventilatory support, suggesting a picture of a healthier population. The proportion receiving LTMV was lower in children with CNS conditions, despite the longer duration of hospital stay at their LTMV initiation (56d, compared to children with muscular myotonies/dystrophy 22d, SMA 3d). This may suggest a complex respiratory situation in these children,^13^ possibly being more multifaceted, somehow underlined by the fact that epilepsy was registered in 58% of the children with CNS conditions, compared to 9% of children with NMDs. Not surprisingly, respiratory tract infections, hypoventilation, and hypoxia were reported before commencing the ventilatory support (Table 2). However, due to missing data, the indications to use LTMV in the present population remain inconclusive.

The study reports the prevalence of MI‐E in a nationwide, neuropaediatric population using several high‐quality national registries. By nature, cough augmentation therapy is a treatment for orphan diseases, and the study has a low number of participants. However, the response‐rate (71%) was high and covered all children with SMA with an MI‐E device for long‐term use in Norway, indicating that the study was representative. That said, being a purely descriptive study, it should not be seen as evidence for the appropriateness of MI‐E treatment in this population.

## Conclusion

The prevalence of children equipped with an MI‐E device in the Norwegian neuropaediatric population was 6 per 1000. Age at initiation and prevalence differed between diagnoses. Long‐term MI‐E use was most common in NMDs, but one‐third of the MI‐E devices were given to children with CNS conditions. Clinical signs of impaired mucus clearance, such as weak and ineffective cough and recurrent respiratory infections were the most common reasons to initiate MI‐E, whereas a prophylactic treatment was mainly reported in children with NMDs. The decision to initiate MI‐E was essentially clinical, as voluntary measurements are unfeasible in the majority of children considered to benefit from MI‐E. Ventilator support was found in more than half of the children using MI‐E.

## Supporting information


**Appendix S1**: Detailed description of the diagnosis‐coding, primary data source, and analyses including age of included children.

**Table S1:** List of ICD‐10 and self‐reported diagnose codes

**Table S2:** Details from child/parent reported indications for and initiation of treatment with mechanical insufflation‐exsufflation

**Table S3:** Clinical characteristics of children using long‐term mechanical insufflation‐exsufflation in Norway and initiation of treatment by regional residency

**Figure S1:** Flow‐chart describing the study progress.

**Figure S2:** Prevalence of long‐term mechanical insufflation‐exsufflation in spinal muscular atrophy by regional residency.

**Figure S3**: Child/parent reported measurements performed to assess respiratory related concerns.

## Data Availability

Restrictions apply to the availability of these data, which were used under license for this study. Data are available from the authors with the permission of the Norwegian Patient Registry and The Norwegian registry for Long‐term Mechanical Ventilation.

## References

[1] Boel L , Pernet K , Toussaint M , et al. Respiratory morbidity in children with cerebral palsy: an overview. Dev Med Child Neurol 2019; 61: 646–53. 30320434 10.1111/dmcn.14060

[2] Proesmans M , Vreys M , Huenaerts E , et al. Respiratory morbidity in children with profound intellectual and multiple disability. Pediatr Pulmonol 2015; 50: 1033–8. 25327770 10.1002/ppul.23114

[3] Tzeng AC , Bach JR . Prevention of pulmonary morbidity for patients with neuromuscular disease. Chest 2000; 118: 1390–6. 11083691 10.1378/chest.118.5.1390

[4] Chatwin M , Ross E , Hart N , et al. Cough augmentation with mechanical insufflation/exsufflation in patients with neuromuscular weakness. Eur Respir J 2003; 21: 502–8. 12662009 10.1183/09031936.03.00048102

[5] Toussaint M , Chatwin M , Gonzales J , Berlowitz DJ , ENMC Respiratory Therapy Consortium . 228th ENMC International Workshop: Airway clearance techniques in neuromuscular disorders Naarden, The Netherlands, 3–5 March, 2017. Neuromuscul Disord 2018; 28: 289–98. 29395673 10.1016/j.nmd.2017.10.008

[6] Chatwin M , Toussaint M , Goncalves MR , et al. Airway clearance techniques in neuromuscular disorders: a state of the art review. Respir Med 2018; 136: 98–110. 29501255 10.1016/j.rmed.2018.01.012

[7] Hull J , Aniapravan R , Chan E , et al. British Thoracic Society guideline for respiratory management of children with neuromuscular weakness. Thorax 2012; 67(Suppl 1): i1–40. 22730428 10.1136/thoraxjnl-2012-201964

[8] Veldhoen ES , Verweij‐van den Oudenrijn LP , Ros LA , et al. Effect of mechanical insufflation‐exsufflation in children with neuromuscular weakness. Pediatr Pulmonol 2020; 55: 510–3. 31909568 10.1002/ppul.24614 PMC7004176

[9] Chatwin M , Bush A , Simonds AK . Outcome of goal‐directed non‐invasive ventilation and mechanical insufflation/exsufflation in spinal muscular atrophy type I. Arch Dis Child 2011; 96: 426–32. 20573738 10.1136/adc.2009.177832

[10] Hov B , Andersen T , Hovland V , Toussaint M . The clinical use of mechanical insufflation‐exsufflation in children with neuromuscular disorders in Europe. Paediatr Respir Rev 2018; 27: 69–73. 29239774 10.1016/j.prrv.2017.08.003

[11] Chatwin M , Simonds AK . Long‐term mechanical insufflation‐exsufflation cough assistance in neuromuscular disease: patterns of use and lessons for application. Respir Care 2020; 65: 135–43. 31690614 10.4187/respcare.06882

[12] Siriwat R , Deerojanawong J , Sritippayawan S , Hantragool S , Cheanprapai P . Mechanical insufflation‐exsufflation versus conventional chest physiotherapy in children with cerebral Palsy. Respir Care 2018; 63: 187–93. 29066586 10.4187/respcare.05663

[13] Blackmore AM , Gibson N , Cooper MS , Langdon K , Moshovis L , Wilson AC . Interventions for management of respiratory disease in young people with cerebral palsy: a systematic review. Child Care Health Dev 2019; 45: 754–71. 31276598 10.1111/cch.12703

[14] Fitzgerald DA , Follett J , Van Asperen PP . Assessing and managing lung disease and sleep disordered breathing in children with cerebral palsy. Paediatr Respir Rev 2009; 10: 18–24. 10.1016/j.prrv.2008.10.003 19203740

[15] Kwon YH , Lee HY . Differences of respiratory function according to level of the gross motor function classification system in children with cerebral palsy. J Phys Ther Sci 2014; 26: 389–91. 24707090 10.1589/jpts.26.389 PMC3976009

[16] Rose L , McKim D , Leasa D , et al. Respiratory health service utilization of children with neuromuscular disease. Pediatr Pulmonol 2018; 53: 1378–86. 30129703 10.1002/ppul.24145

[17] Carter B , Bennett CV , Jones H , et al. Healthcare use by children and young adults with cerebral palsy. Dev Med Child Neurol 2021; 63: 75–80. 10.1111/dmcn.14536 32314347

[18] Rose L , McKim D , Leasa D , et al. Monitoring cough effectiveness and use of airway clearance strategies: a Canadian and UK Survey. Respir Care 2018; 63: 1506–13. 30206128 10.4187/respcare.06321

[19] von Elm E , Altman DG , Egger M , et al. The Strengthening the Reporting of Observational Studies in Epidemiology (STROBE) statement: guidelines for reporting observational studies. J Clin Epidemiol 2008; 61: 344–9. 18313558 10.1016/j.jclinepi.2007.11.008

[20] World Health Organization . The ICD‐10 Classification of Mental and Behavioural Disorders: Clinical Descriptions and Diagnostic Guidelines. Geneva: World Health Organization, 1992.

[21] van den Engel‐Hoek L , Erasmus CE , van Hulst KC , Arvedson JC , de Groot IJ , de Swart BJ . Children with central and peripheral neurologic disorders have distinguishable patterns of dysphagia on videofluoroscopic swallow study. J Child Neurol 2014; 29: 646–53. 24022110 10.1177/0883073813501871

