# Supplementary material for: Prevalence of long‐term mechanical insufflation‐exsufflation in children with neurological conditions: a population‐based study
Source: Dev Med Child Neurol. 2021 Jan 3;63(5):537–44. doi: 10.1111/dmcn.14797 (PMC8048789; doi:10.1111/dmcn.14797)
Supplement: Supplementary file 1 — Appendix S1: Detailed description of the diagnosis‐coding, primary data source, and analyses including age of included children. [file DMCN-63-537-s003.docx]

# Method

## The population

The population of interest was defined as children with a main- or bi-diagnosis in the International Classification of disease, version 10 (ICD-10) Chapter G; “Diseases of the nervous system”. To include hypotonic children with metabolic conditions, main diagnosis from chapter E 70-90; “Metabolic disorders” were correspondingly registered.

## The coding of diagnoses

If more than one diagnose of interest were listed by the Norwegian Patient Registry (Registry #2) the latest recorded main diagnosis in ICD-10, Chapter G were used for analysis.

## Estimation of the neuro-paediatric population

The following methodical choices were made:

- If the aggregated tables provided from the Norwegian Patient Registry reported cells as <5 (to maintain anonymity), <5 was replaced with 1. This may have led to an underestimation of the neuro-paediatric population, especially in rare conditions. The maximal error is 0.5% (if the true value of <5 is 4).

- If more than one main diagnosis in ICD-10, Chapter G were listed at the last recording, the individual is counted in both diagnosis groups. If so, a slightly overestimation of the neuro-paediatric population is possible.

- If a child holding a MI-E device at the last recording was listed in two diagnosis groups by the Norwegian Patient registry, the patient-reported diagnosis was used as the main diagnosis.

The children were divided in two main groups based on the origin of their main diagnose (E-table-1). To maintain anonymity for individuals with rare diagnoses (<5), four children with a main diagnose classified as metabolic disorder (ICD-10; E70-90) and one with peripheral nerve disease (ICD-10; G60-64) were included as CNS-disorder as they all had a CNS-condition as bi-diagnosis.

## The completeness of the primary data-source:

The primary data-source were the “Departments for Medical Home-Care Equipment” located at 18 hospitals throughout the country that provided a record of children with a given device for mechanical cough. The list was reviewed by personnel responsible for the children’s pulmonary follow up.

Children immigrating to Norway with a device would be listed in the registers of “Department for Medical Home-Care Equipment” as these departments are responsible for providing necessary accessories without cost (mask and tubing) and also free service for the devices.

Private purchase appears unlikely due to the high cost of the MI-E equipment and the provision for free by the Norwegian government if prescribed by a specialized doctor.

The company (Philips Respironics) selling devices in Norway have confirmed (private communication per e- mail, august 2020) that private sale including internet sale is not offered in Norway.

For the children receiving concomitant ventilatory support the completeness of the primary data source was confirmed by Registry #4; The Norwegian quality registry for LTMV as users of MI-E devices was reported independently by this registry.

## The age:

Age at prevalence day (Aug 15^th^ 2017) is used when calculation age in Study-population 1. Due to lag in time between identifying the possible sample (Feb-March 2017) and the return of questionnaire (during 2018), there was approximately one year of displacement in age.

Age at first establishing the primary data source (April 1^st,^ 2017) is used for calculations including age in Study-population 2.
