# Supplementary material for: Prevalence of long‐term mechanical insufflation‐exsufflation in children with neurological conditions: a population‐based study
Source: Dev Med Child Neurol. 2021 Jan 3;63(5):537–44. doi: 10.1111/dmcn.14797 (PMC8048789; doi:10.1111/dmcn.14797)
Supplement: Supplementary file 2 — Table S1: List of ICD‐10 and self‐reported diagnose codes [file DMCN-63-537-s007.docx]

E-table 1: Diagnose and sub-groups with ICD-10 codes and child/parent reported diagnosis-name.

| **Diagnose-group** | **ICD-10 code** | **Sub-group** | **Child/Parent**  **diagnosis names** |
| --- | --- | --- | --- |
| Neuro-muscular disorders | G12 | Spinal muscular atrophy /  Neuromuscular junction | SMA1-3  SMARD  Congenital myopathy  Nemalin myopathy  Central Core myopathy  Duchenne muscular dystrophy  Limb Girdl dystrophy  Emery dreyfuss dystrophy  Merosin-deficit |
|  | G60_G64 | Peripheral nerve |  |
|  | G70_G72 | Muscular dystrophies  /myopathies |  |
| Central nervous system | G00_G09 | Encephalitis | Cerebral Palsy  Congenital varicella syndrome  Lissen encephalopathy  MEGDEL syndrome  Metachromatic leukodystrophy (MLD) Methylmalonic acidemia with homocystinuria (MNA) Migrating Partial Seizures/Epilepsy in Infancy (MPEI)  Myelitis  Myomeningiocele  Partially trisomy  Sept optic dysplasia  Muco-poly-saccharidosis (MPS)  Progressive  encephalitis |
|  | G30_G32 | Degenerative conditions in CNS |  |
|  | G80_G83 | Cerebral Palsy |  |
|  | G90_G99 | Other conditions in CNS |  |
|  | E70_E90 | Metabolic disorders |  |

E-table 1: Diagnosis (main and sub-groups) according to the International Classification of Disease, version 10 (ICD-10), with diagnosis names and child/parent reported diagnosis.
