# Supplementary material for: Prevalence of long‐term mechanical insufflation‐exsufflation in children with neurological conditions: a population‐based study
Source: Dev Med Child Neurol. 2021 Jan 3;63(5):537–44. doi: 10.1111/dmcn.14797 (PMC8048789; doi:10.1111/dmcn.14797)
Supplement: Supplementary file 3 — Table S2: Details from child/parent reported indications for and initiation of treatment with mechanical insufflation‐exsufflation [file DMCN-63-537-s001.docx]

E-table 2: Details about initiation of the long-term MI-E treatment.

| Diagnosis  “Study population 2” (n) | Total  73 | Neuro Muscular Disorders  47 | | Central Nervous System  26 | P-value |
| --- | --- | --- | --- | --- | --- |
|  |  | **Spinal muscular atrophy**  24 | **Muscular**  **dystrophies/**  **myopathies**  23 |  |  |
| Q: Why did you start MI-E?  Total answers (n; %)  - To prevent respiratory infections  - Weak cough resulting in problems moving secretions/frequent respiratory infections  Q: In what context did you initiate the long-term MI-E treatment?  Total answers (n; %)  - During admission for RTI  - During admission for other  - Elective in outpatient clinic  - Do not remember  Q: Who performed the education and training?  Total answers (n; %)  - Physician  - Physiotherapist  - Physician, Physiotherapist and nurse | 70  17; 24%  53; 76%  69  29; 42%  21; 31%  14; 20%  5; 7%  47  3; 6%  30; 64%  14; 30% | 23  5; 22%  18; 78%  23  13; 57%  4; 17%  2; 9%  4; 17%  13  0  8; 62%  5; 38% | 22  10;45%  12; 55%  22  8; 36%  10; 45%  4; 18%  0  18  0  14; 78%  4; 22% | 25  2; 8%  23; 92%  24  8; 33%  7; 29%  8; 33%  1; 4%  16  3; 19%  8; 50%  5; 31% | 0.011  0.037  0.011 |

E-table 2. Details about the initiation of the long-term use of Mechanical Insufflation-Exsufflation reported by child/parent. Abbreviations: MI-E = Mechanical insufflation-exsufflation, RTI= Respiratory tract infections. P-values are referring to difference of answers by diagnose-groups using Chi-square test for contingency tables (Fischer exact test).
