# Supplementary material for: Prevalence of long‐term mechanical insufflation‐exsufflation in children with neurological conditions: a population‐based study
Source: Dev Med Child Neurol. 2021 Jan 3;63(5):537–44. doi: 10.1111/dmcn.14797 (PMC8048789; doi:10.1111/dmcn.14797)
Supplement: Supplementary file 4 — Table S3: Clinical characteristics of children using long‐term mechanical insufflation‐exsufflation in Norway and initiation of treatment by regional residency [file DMCN-63-537-s005.docx]

Table S3: Clinical characteristics of children using long-term MI-E in Norway and initiation of treatment by regional residency.

| Health-region | n | Overall | South-East  n=57 | West  n=9 | Middle  n=5 | North  n=2 | p-value |
| --- | --- | --- | --- | --- | --- | --- | --- |
| Continuous data: median (min-max) |  |  |  |  |  |  |  |
| Age (years months)  Age at MI-E initiation*  MI-E use duration at study* | 73  70  70 | 10y 1 mo  (1y5m-17y9m)    5y (0 – 15)  2y 6 mo | 10y 4 mo  (1y5m-17y1m)  6 (0-15)  3y 6 mo | 10y 6 mo  (1y7m-17y11m)  4 (0-12)  5y 2mo | 4y 1mo  (1y8m-6y9m)    1.5 (0-3.5)  2y 2mo | 10y 5mo  (9y4m-11y6m)  3 (1 – 5)  7y 5mo | **0.047**  0.202  0.268 |
| Categorical data; n |  |  |  |  |  |  |  |
| Diagnosis-group  NMD  CNS | 73 | 47  26 | 37  20 | 5  4 | 5  0 | 0  2 | 0.082 |
| Gender  Male  Female | 73 | 42  31 | 34  23 | 4  5 | 3  2 | 1  1 | 0.851 |
| Q: Why did you start MI-E?) *  - To prevent respiratory infections  - Weak cough resulting in problems removing secretions/frequent RTIs | 70 | 17  53 | 13  41 | 3  6 | 1  4 | 0  2 | 0.779 |
| Q: In what context did you initiate the long-term MI-E treatment? *  - During admission for RTI  - During admission for other  - Elective in outpatient clinic  - Do not remember | 69 | 29  21  14  5 | 20  19  11  3 | 5  1  1  2 | 3  1  1  0 | 1  0  1  0 | 0.517 |
| Long-term  Mechanical ventilation **  User  Non-user | 73 | 41  32 | 30  27 | 7  2 | 3  2 | 1  1 | 0.561 |

E-table 3: The clinical characteristics of the participants (Study-population 2) according to regional residency. Abbreviations: MI-E: Mechanical insufflation-exsufflation, RTI= Respiratory tract infections. P value is difference between groups calculated by Kruskal-Wallis rank test when continuous data or Chi-square test for contingency tables when categorical data. P < 0.05 marked in bold. *data from questionnaire. **data from National registry for Long-term Mechanical Ventilation.
