# Supplementary figures and images for: Prevalence of long‐term mechanical insufflation‐exsufflation in children with neurological conditions: a population‐based study
Source: Dev Med Child Neurol. 2021 Jan 3;63(5):537–44. doi: 10.1111/dmcn.14797 (PMC8048789; doi:10.1111/dmcn.14797)

# Patient collection and study progress

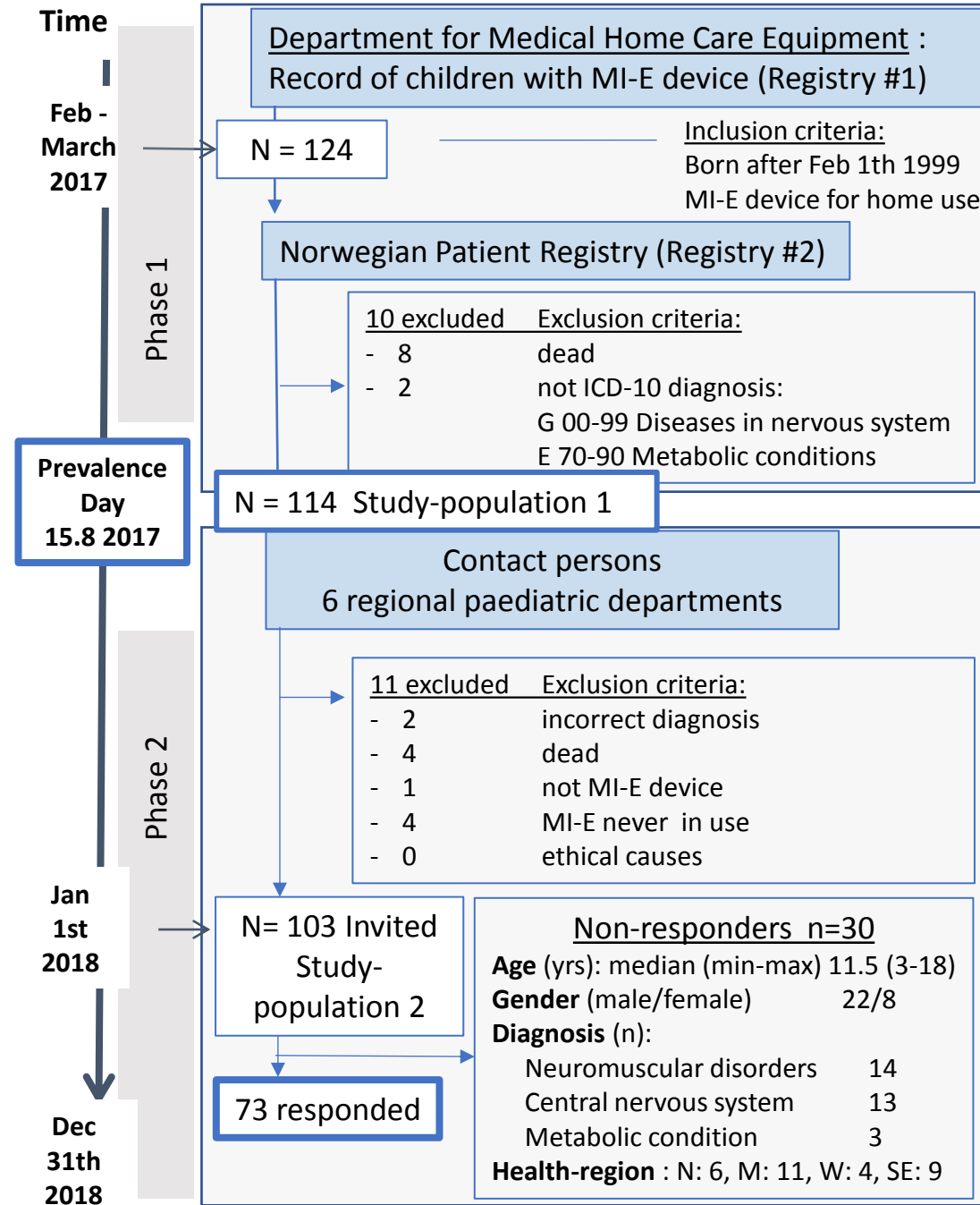

Supplement: Supplementary file 5 — Figure S1: Flow‐chart describing the study progress. [file DMCN-63-537-s004.pdf]

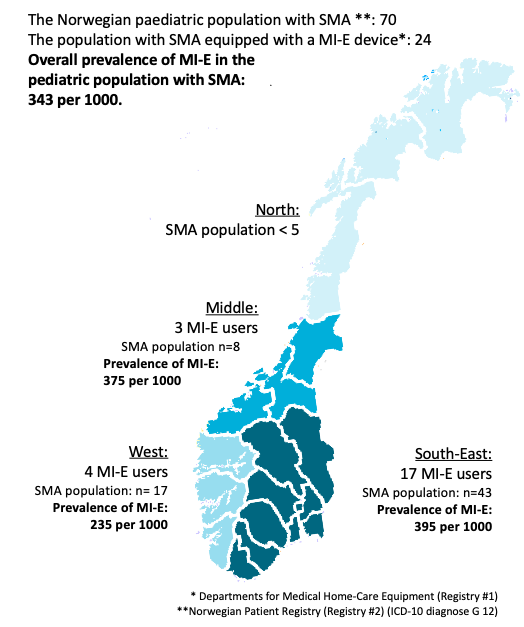

Supplement: Supplementary file 6 — Figure S2: Prevalence of long‐term mechanical insufflation‐exsufflation in spinal muscular atrophy by regional residency. [file DMCN-63-537-s002.png]

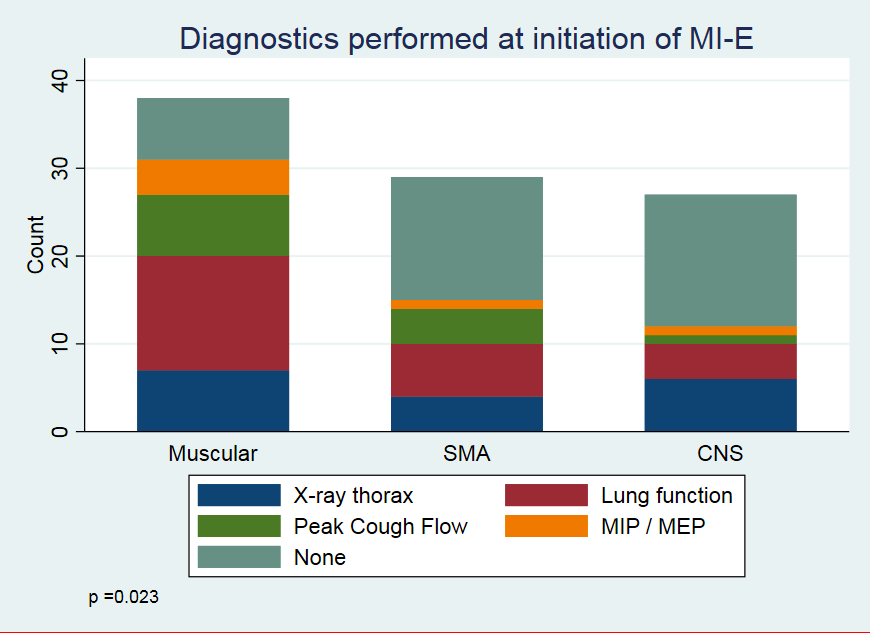

Supplement: Supplementary file 7 — Figure S3: Child/parent reported measurements performed to assess respiratory related concerns. [file DMCN-63-537-s006.png]
